# Dr. Kinglake, on Arthritis

**Published:** 1803-02-01

**Authors:** Robert Kinglake

**Affiliations:** Taunton


### *To the Editors of the Medical and Physical Journal.*

GENTLEMEN,

WHEN I last addressed the public through the medium of your Journal,* on the salutary effects resulting from the topical application of cold water in arthritic affection, the practice was new in my experience; and (as was observed

---

* No. xxxiii. p. 454.

served in that paper) a theoretical view of the analogy subsisting between gouty, and every other description of inflammation, led me to make trial of its powers. Although popular prejudice against the mode of remedy is too insuperable to admit of so extensive an application of it as is necessary to bring it into vogue; yet particular instances have lately occurred in my practice, which confirm very satisfactorily, my former opinion of its superior curative power. Amidst a variety of cases, in which the topical reduction of temperature rendered much temporary alleviation, but in which, a distrust in its safety too much interrupted its use, to afford any decisive testimony on the subject, are the subsequent examples of its salutary efficiency, in which, its employ was conducted in a manner that warrants placing the results on the records of medical facts.

Case I. A gentleman who had repeatedly suffered under severe paroxysms of gout, experienced a few months since, a recurrence of the affection. It shewed itself as usual, on the bulb of the great toe, which was much swollen, highly inflamed, and exquisitely painful. The local disease soon extended its stimulant influence over the system, inducing violent symptoms of general irritation. The topical inflammation proceeded in the accustomed manner during about a fortnight, when my advice was desired. On enquiry, it appeared that the routine treatment of covering the affected part with flannel had been sedulously observed; confinement to bed in a warm room, and a liberal use of spirituous liquors, were also judged expedient, to obviate every risk of repulsion. By this course, the patient had added to the local establishment of the disease, considerable constitutional debility. No circumstance in the case gave me the smallest concern for the safety of employing very freely reduced temperature, which was applied by means of cloths wetted in an aqueous fluid. This was directed to be repeated every half hour, or even oftener, if a painful degree of heat should sooner prevail. Sudorific medicine, composed of camphorated tincture of opium (tinctura opii camphorata) and ammoniated tincture of guaiacum (tinctura guaiaci ammoniata) in doses of two drachms each, diluted with water, was likewise ordered to be taken at intervals of six hours. Instantaneous relief arose from the cold application, but which lessened with returning heat, and was promptly renewed by the repetition of the remedy.

The intolerable pain with which arthritic inflammation is accompanied, was so perfectly controlable in this case by reduced temperature, that the patient kept it so constantly diminished by its frequent use, as very speedily to subdue it altogether. On the second day of its employ, no disposition to return of pain on lengthening the intervals of recurring to it continued. The swelling and redness were also much decreased; a livid hue indeed had superseded the florid aspect. The peculiar stiffness arising from debility, and probably from more or less of serous effusion in the cellular texture of the inflamed part, prevented either facility or strength of motion for several days; but with the local disease terminated the systematic irritation, and every other complaint.

The patient waited on me at the distance of several miles in the course of a week, to shew the progress of his recovery, when bark, a nutritious diet, friction, and bandage on the affected limb, were farther directed. He soon after was enabled to resume his usual active avocations, without any inconvenience. This was a case of sheer gout, unmixed with any of those anomalies, or irregularities, which often obscure its evident character. The efficacy of the treatment rested on the anti-inflammant power of topical cold. The remedy was prompt, progressive, and unequivocal.

The design of the medicine here employed was, by its diffusive exciting quality, to calm and harmonize the motive powers, to proportion duly the distribution of the circulating fluids, to balance the various secretions, and thereby counteract either the formation or continuance of any sympathetically morbid action which the pain of protracted gout is liable to occasion; but even this indication for the use of medicine, is founded rather on extreme caution, than an unquestionable necessity.

Cases are in my recollection, in which the prejudices of theory would have shuddered to have hazarded a patient without a medicinal guard, but in which none was employed; and the event proved that none was requisite.—One of this description is the following:

Case II. A gentleman who had, on the slightest catarrhal affections from exposure to change of temperature, been for many years subjected to podagral inflammation. The great toe of one or both feet had been invariably the seat of the complaint. He was lately attacked on a journey, so as to be disabled from prosecuting it.

Having

Having learnt the good effect of my mode of treating gout, my advice was desired. The unprejudiced correctness of the patient's understanding, induced him confidently to submit to the full force of reduced temperature.

Cloths wetted in cold water were first applied to the affected part in the evening, and renewed frequently throughout the ensuing night. The morbid heat yielded before the morning, and with that also the tumefaction, redness, and distempered sensibility. A sense of numbness, or rather weight, only was felt in the morning, which was no obstacle to the patient's pursuing his journey. Cold application was continued for a day or two as a preventive. No farther complaint occurred, and the patient has not, to my knowledge, suffered from arthritic affection since that time. No medicine was directed. Every thing that tended to heat by stimulating was prohibited, and nothing more appeared to be necessary.

This is one among the numerous cases of rapid removal of arthritic inflammation by reduced temperature, which would perhaps almost constantly occur, were similar practice to be uniformly adopted. The earlier the mode of treatment is resorted to, the more speedily would the inflammatory affection be subdued, and consequently the danger (if any) of the irritation being transferred to any other part be proportionately less, as sufficient time would not then be afforded for any associated or sympathetic movements to be generated, either generally or partially, in the system, which might possibly assume a similar action on the cessation of the primary disease.

The advantages also resulting from an early check being given to the disorder, consist in obviating change of structure in the vascular fabric of the affected parts, arising either from the generation of new vessels, the decomposition of old, or from inorganic and obstructing effusions in the cellular texture.

Case III. A gentleman, whose constitutional health had been long subjected to an habitual recurrence of podagral malady at uncertain intervals, and who had been lately benefitted by the topical employ of diminished temperature under my direction, desired my advice in the earliest stage of an attack; so early indeed, as to be able to ride a distance of several miles to consult me. The great toe of one foot was then rapidly tumefying, highly heated, and becoming acutely painful; the foot and knee-joints also partook of the irritation. The affected extremity was ordered to be enveloped in a cloth dipped in a cold fluid consisting chiefly of water, but unimportantly disguised both to obviate the probable alarm of the patient, and the *certain dread* of the family apothecary, who had distinguished himself as a very *Vulcan*, by his *fiery ordinances*, in the treatment of gout.

The cold application allayed, in a few hours, the inflammatory symptoms, and its continuance speedily re-conducted the disordered action to the natural motive conditions of ease and health. This patient had never before a fit of the gout that did not confine him for several weeks; and if it be allowable to presume from the incipient violence of the last attack, its progressive and permanent severity would have been at least equal to what had been formerly experienced. Its sudden repression, and speedy removal, therefore, evince, in the most satisfactory manner, that arthritic inflammation is indubitably a disease of excessive temperature; that its natural antidote is diminished heat; that this remedy is conveniently applied through the medium of cold water; and that probably the most prompt and efficacious mode of employing it, would be by either immersing the affected limb into that fluid, or by incessant affusion with it, until the painful sense of morbid heat should wholly subside.

My experience fully warrants me in believing that this effect would almost invariably happen in the course of a few hours.

Case IV. A middle aged man, by trade a mechanic, had been during several years periodically afflicted with gout. It usually attacked the feet, and, occasionally, also the hands. His accustomed confinement in this disorder, disabled him for a considerable length of time from engaging in his mechanical employment. In his last seizure (but a few weeks since) he desired my advice. The plan of treatment pursued in the preceding cases was commenced. The affected parts were assiduously plyed with cold water, to which (as in every other instance) the pain speedily yielded; but if the renewal of cold was delayed beyond half an hour, distempered heat would return, accompanied, as the patient conceived, with an aggravated degree of irritation.

The fluid, which was topically applied, was so slightly disguised, that it appeared too much like simple water not to awaken the patient's suspicion, and consequently his fear of its noxious repelling quality. This dread, by suitable

-able explanation, however, was soon allayed, and a more unremitted continuance of cold application to the affected parts, completely subdued in a few days the several inflammations, leaving only the usual sense of debility and numbness, for which he was directed to use brisk friction with a flesh brush, two or three times a day. The camphorated tincture of opium (tinctura opii camphorata) and ammoniated tincture of guaiacum, (tinctura guaiaci ammoniata) in doses of two drachms each, in an aqueous vehicle, were also taken every four hours, for the purpose of quieting the prevailing systematic irritation, and determining to the surface; an effect strongly indicated, to remedy an unvaried dryness of the skin.

The patient called on me about a week after the cessation of his complaints, and discontinuance of all medicinal treatment, thanking me for the aid he had received, adding, that he had never before been freed from a fit of the gout so rapidly, with so little pain, and with so early a return of the natural powers of the affected parts.

This case afforded me an opportunity of remarking with much satisfaction, the perfect safety of reducing the inflammatory temperature of gouty affection, even in the worst state of constitutional health. A consumptive tendency was strikingly discoverable in the feebleness with which the ordinary functions of life were performed in this patient. Destitute of arterial tone and muscular vigour, and with that peculiarly dry unyielding cough which characterises tubercular irritation, the reactive powers of the system were extremely weak, and must have been overwhelmed, if, according to popular prejudice, the sudden allaying of arthritic inflammation could ever be followed by a morbid transference of it to some vital part of the system. This idea is really a mere bugbear, occasionally sanctioned even with the solemn gravity of medical erudition, which, has served to alarm the prejudiced and the ignorant in every age from remote antiquity, and to preclude the adoption of a rational mode of cure. The reverse of the prevailing opinion is true. It is not the early extinction of gouty inflammation that endangers a retrocession of its morbid influence on the system, but its being protracted on a part.

When by long continuance a local establishment of the disease is formed, it will ultimately diffuse its distempered action over the frame, and thus by either generally or partially undermining the motive powers of health, become more or less predominant, according to circumstances of tempertemperamental atony, and susceptibility for morbid impression.

Case V. This is an instance which may serve, *instar omnium*, as a decisive proof of perfect security in employing reduced temperature in external inflammation, whether purely arthritic, or as the *blending acumen* of the Nosologist would denominate, rheumatismo-arthritic, or any other description of inflammatory affection. The subject of this case was a labouring man, who had repeatedly suffered from careless exposure to cold, during the refrigerating influence of profuse perspiration. On a former occasion, severe peripneumony resulted from an abrupt reduction of temperature; in the instance under consideration, a violent inflammatory affection of every joint of both the hands and feet was the consequence. The shining tumid aspect, and intolerable pain peculiar to the gout, induced a suspicion of its being that malady; but in the excessive temperature that prevailed, that distinction to me was no more than nominal. When inflammation is accompanied with a sense of heat literally burning, it is very unimportant what it is called; it is of more moment to repress its hurtful tendency with the utmost promptitude.

This patient's limbs were immmediately enveloped with cloths dropping with cold water, which were renewed about every half hour. The moisture was detached by evaporation from the cloths, as rapidly as if held before a fierce fire. The pain, as always happens, was speedily assuaged, but soon recurred, if not prevented by a timely renewal of cold; the constant application of which, subdued by the following day the irritation of the feet and hands; when associated or sympathetic pain began to rage on the knees and shoulders, those parts soon became as inflamed and motionless as those which the irritation had relinquished. The same treatment was pursued, and with similar effect. The cessation of pain in these newly affected parts, was the recommencement of it in those which were first diseased; thus did the irritation alternately change its position during several weeks. All this time the patient may be said to have been *soaked* in cold water.

The pain was tolerable (scarcely indeed deserving the name) when the remedy was freely used; but either inattention or fear of its proving mischievous, occasionally interrupted its due employ, to which probably the want of more early and decisive efficacy may be attributed. Latterly it was judged right not only to reduce the heat of the parts parts actually inflamed, but also to keep those which had been recently relieved, in a state of diminished temperature. This expedient perfectly succeeded; no farther inflammation happened; and from that hour the alternate shifting of the affection ceased, and the patient began to recover his constitutional strength. No serious ailment occurred, at any stage of the disease, to the general health; nor did the occasional interposition of purgative, sudorific, and narcotic medicine, in any degree mitigate the severity of pain.

Although a long series of recurring inflammation had broken and exhausted the constitutional strength, inducing that morbid state of irritability, which may be supposed to be peculiarly favourable to admitting diseased impression, yet nothing like a transposition of the disordered excitement happened to any internal part of the system, and even under circumstances in which associative or sympathetic motions seemed to be performing the reciprocal office of *derivants* of irritation from the several affected parts.

It would be difficult to adduce an example more strong than this, in testimony of the curative power of diminished heat in the worst conditioned instances of inflammation. It evinces, beyond all doubt, that excessive temperature is the *proximate cause,* and not merely a *symptom* or *effect* of inflammation. How this is generated, can only be known by investigating these distempered conditions of motive power, that occasion its morbid evolution and redundancy. This enquiry has long been with me a favourite speculation, and may probably hereafter, if leisure should permit, be sufficiently matured to warrant my submitting it to public consideration.

Subjoined are two letters addressed to me on the subject of this paper. The testimony contained in these communications, contributes to verify the correctness of my observation in both these and my former cases on the cure of arthritic inflammation, and may be justly considered as a valuable appendage to them.

I am, &c.

*Taunton, Dec.* 22, 1802. ROBERT KINGLAKE.

